# Optimal Conditions for Continuous Immobilization of *Pseudozyma hubeiensis* (Strain HB85A) Lipase by Adsorption in a Packed-Bed Reactor by Response Surface Methodology

**DOI:** 10.1155/2012/329178

**Published:** 2012-01-23

**Authors:** Roberta Bussamara, Luciane Dall'Agnol, Augusto Schrank, Kátia Flávia Fernandes, Marilene Henning Vainstein

**Affiliations:** ^1^Centro de Biotecnologia, Universidade Federal do Rio Grande do Sul, Porto Alegre 91501-970, RS, Brazil; ^2^Laboratório de Química de Proteínas, Departamento de Bioquímica e Biologia Molecular, Universidade Federal de Goiás, Goiânia 74001-970, GO, Brazil

## Abstract

This study aimed to develop an optimal continuous process for lipase immobilization in a bed reactor in order to investigate the possibility of large-scale production. An extracellular lipase of *Pseudozyma hubeiensis* (strain HB85A) was immobilized by adsorption onto a polystyrene-divinylbenzene support. Furthermore, response surface methodology (RSM) was employed to optimize enzyme immobilization and evaluate the optimum temperature and pH for free and immobilized enzyme. The optimal immobilization conditions observed were 150 min incubation time, pH 4.76, and an enzyme/support ratio of 1282 U/g support. Optimal activity temperature for free and immobilized enzyme was found to be 68°C and 52°C, respectively. Optimal activity pH for free and immobilized lipase was pH 4.6 and 6.0, respectively. Lipase immobilization resulted in improved enzyme stability in the presence of nonionic detergents, at high temperatures, at acidic and neutral pH, and at high concentrations of organic solvents such as 2-propanol, methanol, and acetone.

## 1. Introduction

Biocatalyst enzymes play an important role in biotechnological applications due to their extreme versatility with respect to substrate specificity and stereoselectivity and exhibit many other features that render their use advantageous when compared to conventional chemical catalysts. As an example, fat and oil hydrolysis using NaOH as a catalyst requires high pressure and temperature to achieve high efficiency (97-98%); in contrast, the same process can be carried out effectively at normal temperature and pressure using lipases, with significant decrease in wastewater production. Lipases (triacylglycerol acylhydrolase; EC 3.1.1.3) catalyze the hydrolysis of triglycerides to glycerol and fatty acids, as well as a variety of reactions in nonaqueous medium (e.g., transesterification, esterification, and interesterification). Such enzymatic properties allow a series of biotransformation reactions that lead to multiple industrial applications in foods, flavors, pharmaceuticals, detergent formulation, oil/fat degradation, cosmetics, and environmental remediation [1–4].

However, soluble enzymes usually exhibit lower stability than chemical catalysts and often cannot be recovered and reused. This severely hinders their application in practice. Nevertheless, this problem can be overcome by enzyme immobilization, which enhances thermal and operational stabilities, ease of handling, and prevention of aggregation and autolysis. Besides, immobilized lipases (IE) on solid support allow recoverability and reuse thus significantly reducing operational costs of industrial processes [4–8].

An immobilization process involving hydrophobic binding of lipases by adsorption has proved success due to the enzyme affinity for water/oil interfaces [6, 9, 10]. Enzyme adsorption onto hydrophobic solid surfaces is assumed to involve the large hydrophobic area that surrounds the lipase active site, so lipases are believed to recognize these solid surfaces similarly to their natural substrates and suffer interfacial activation during immobilization [9–11]. The high activation of lipases upon immobilization, the possibility to associate the immobilization with the purification of lipases, the low activity loss of the adsorbed enzymes in organic environment, and the strong but reversible immobilization that enables support recovery are factors that make this simple method cost effective [6, 9, 11, 12].

Immobilization of lipases can be achieved either by batch or continuous reactors, such as packed-bed reactors. The latter are currently preferred over the former due to speed and ease of operation, low investment, and reduced loss of the solid support in the process [13].

The main objectives of this work were to develop an optimal continuous process for lipase immobilization and to compare the immobilized and free lipase from *P. hubeiensis* (strain HB85A). For immobilization, relationships between variables (immobilization time, immobilization pH, and enzyme/support ratio) and the response (IE activity) were analyzed by response surface methodology (RSM) and factorial experimental design.

## 2. Materials and Methods

### 2.1. Materials

Commercially available polystyrene-divinylbenzene support (DIAION HP-2013605-EA) and *p*-nitrophenylpalmitate (*p*-NPP, N2752-1G) were purchased from Sigma-Aldrich (St. Louis, USA). All other chemicals were of analytical grade.

The lipase-producing yeast *P. hubeiensis* (strain HB85A) was originally isolated from the phylloplane of *Hibiscus rosa-sinensis *(Farroupilha Park, Porto Alegre, RS, Brazil). In our previous work, this strain was phenotypically characterized by standard morphological and physiological tests and the identification was confirmed by sequencing of the D1/D2 region of the 26S rDNA (GenBank access number DQ 123912) [14]. Lipase was produced in a batch culture of *P. hubeiensis*, carried out in a 14 L New Brunswick MF 14 Bioreactor with 10 L basal medium (glucose 2.0 g/L, peptone 5.0 g/L, MgSO_4_ 0.1 g/L, K_2_HPO_4 _1.0 g/L) and 20 g/L of soy oil as enzyme inducer. Standard operation conditions were agitation rate of 200 rpm at 28°C with an airflow rate of 1 vvm and a 24 h fermentation time, without pH control. Cells were removed by centrifugation at 10.840 *g* for 10 min, and the culture supernatant was used as the enzyme source. A lipase activity of 1200 U/L at pH 8.0 and a protein concentration of 25 mg/L were detected in the culture supernatant. No protease activity was found [14]. This culture supernatant is hereafter referred to as free lipase culture supernatant (FLCS).

### 2.2. Immobilization of Lipase by Adsorption in a Packed-Bed Reactor

**Figure 1:**
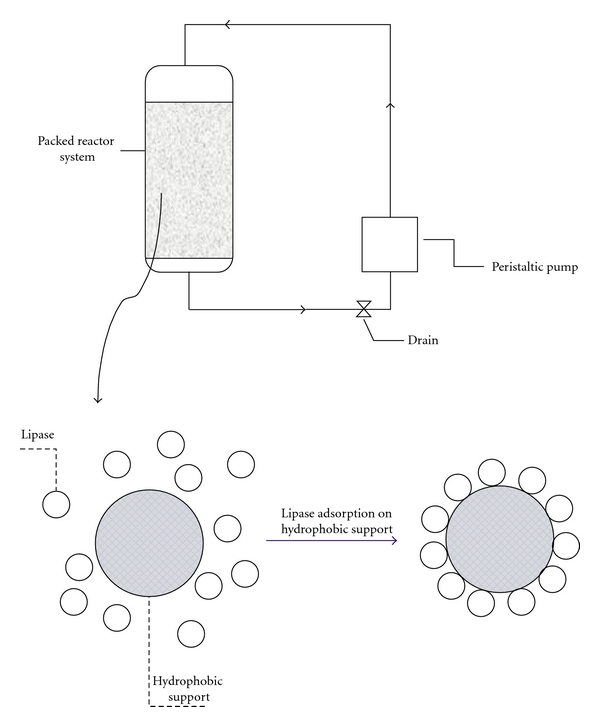
Schematic diagram of the support and of the lipase adsorption process.

Lipase was immobilized onto the hydrophobic resin polystyrene divinylbenzene (matrix: styrene divinylbenzene, particle size: 250–850 *μ*m, pore volume: 1.30 mL/g, pore size: 260 A°) by adsorption (Figure 1). The optimization of immobilization was studied using response surface methodology (RSM) and central composite rotatable design (CCRD) 2^3^ plus axial and central points. The factors assessed were immobilization time (*t*: 1, 60, 150, 240, 300 min), immobilization pH (pH: 1.0, 3.0, 5.0, 7.0, 9.0), and enzyme/support ratio (ES: 1, 405, 1000, 1600, 1999) (Table 1). The IE activity/g of solid support was studied as the response.

A typical immobilization procedure was executed: the polystyrene-divinylbenzene support (1.0 g) was packed into a glass column (Ø 2.5 cm  × 20 cm); in the packed reactor system, a peristaltic pump was used to recycle the solutions used at a flow rate of 2 mL/min. The support was pretreated, as recommended by the supplier, in cycles of 15 min with 10 mL of distillated water followed by 10 mL of buffer solutions to equilibrate the system for the immobilization reaction. Afterwards, the FLCS was added to the column, at 25°C, and cycles of different time intervals were done to immobilize lipase by adsorption. Unbounded lipase was then drained out of the column, and the support was washed three times with 2.5 mL of buffer solution/g of solid support at the studied pH values. The washing buffers were tested for lipase activity in order to ensure that all unbounded lipase was drained out of the column. To determine the amount of lipase immobilized on the support, an aliquot of this matrix (100 mg) was used to assess lipase activity as described in Section 2.3. Pretreated support without immobilized enzyme was used as a control.

### 2.3. Lipase Activity Spectrophotometric Assay

The assay was performed by measuring the increase in absorbance at 410 nm in a visible spectrophotometer (Ultrospec 2000) caused by the release of *p*-nitrophenol after hydrolysis of *p*-nitrophenylpalmitate (*p-*NPP) at 37°C for 30 min, with reference to a control without enzyme. To initiate the reaction, 0.1 mL of the FLCS or 100 mg of the support with the IE was added to 0.9 mL of substrate solution containing 3 mg of *p*NPP dissolved in 1 mL 2-propanol and 9 mL of reaction mixture (40 mg of Triton X-100, 10 mg of Arabic gum dissolved in buffer solution) [15–17]. The activity of the immobilized enzyme was measured in the low-density solution at 410 nm, after sedimentation by gravity. One unit of lipase (U) was defined as the amount of enzyme that releases 1 *μ*mol *p*-nitrophenol/h in the assay conditions described previously. The calibration curve was prepared using *p*-nitrophenol as the standard (100 *μ*mol/mL).

### 2.4. Free and Immobilized Lipase Characterization

Lipase characterization was performed using the FLCS and the IE. Conditions for lipase activity evaluation were the same as described previously (Section 2.3) unless stated otherwise.

#### 2.4.1. Effect of Temperature and pH on Lipase Activity

Two experimental designs using RSM and CCRD 2^2^ were utilized to optimize temperature (*T*: 30, 36, 50, 64, 70°C) and pH (pH: 3.0, 4.0, 6.0, 8.0, 9.0) of reaction. The FLCS and the IE activities were the dependent variables studied as the response; their levels are presented in Table 2.

### 2.5. Stability Parameters

#### 2.5.1. Effect of Temperature and pH on Lipase Stability

The lipase temperature stability was determined by incubating 100 *μ*L of the FLCSs or 100 mg of the IE for 2 h at 30, 40, 50, 60, and 70°C in the absence of substrate. Relative activity was measured by the spectrophotometric assay (Section 2.3) under optimized reaction conditions for the FLCS (pH 4.6 at 68°C) and the IE (pH 6.0 at 52°C). The hydrolytic activity of the control enzymes, kept for 2 h at room temperature (25°C), was taken to be 100%.

The lipase pH stability was determined by incubating 2 *μ*L of FLCS or 2 mg of IE with 98 *μ*L or 100 *μ*L, respectively, of buffer solutions (pH 3.0, 4.0, 5.0, 6.0, 7.0, 8.0, and 9.0) for 2 h at 50°C in the absence of substrate. Relative activity was measured by the spectrophotometric assay (Section 2.3) under optimized reaction conditions. The control was done as before.

#### 2.5.2. Effect of Detergents and Diverse Chemicals on Lipase Activity

In order to analyze detergent and chemicals effect on lipase activity, 2 *μ*L of the FLCS diluted in 98 *μ*L of 50 mM citrate-phosphate pH 7.0 and 2 mg of the IE diluted in 100 *μ*L of the same buffer were incubated for 1 h at 50°C in the presence of 1% (v/v) of detergents (Triton X-100, Tween 80, Tween 20, and SDS) and 5 mM of BaCl_2_, CaCl_2_, MgCl_2_, KCl and EDTA. As a control, 2 *μ*L of the FLCS or 2 mg of the IE were incubated with the buffer solution in the absence of chemicals for 1 h at 50°C. Relative activity was measured by the spectrophotometric assay (Section 2.3) under optimized reaction conditions. The hydrolytic activity of the FLCS and the IE without the addition of any substance was taken to be 100%.

#### 2.5.3. Lipase Stability in Organic Solvents

The FLCS and the IE were incubated in 50 *μ*L of organic solvents (acetone, methanol, ethanol, 2-propanol, and butanol) at different concentrations (20, 50, and 80% v/v) for 1 h at 50°C. As a control, the FLCS and the IE were incubated with the buffer solutions without organic solvents for 1 h at 50°C. Relative activity was measured by the spectrophotometric assay (Section 2.3) under optimized reaction conditions. The control was done as above.

#### 2.5.4. Storage Stability

The FLCS and the IE were stored at 4°C. Enzyme stability was tested for a period of 40 days by the spectrophotometric assay (Section 2.3) under optimized reaction conditions. The hydrolytic activity of the fresh enzyme was taken to be 100%.

### 2.6. Statistical Analysis

Statistical treatment of immobilization conditions and reaction optimization was performed by multivariate analysis. Results were analyzed using the software STATISTICA 7.0 (Statsoft Inc. 2325 East 3rd Street, Tulsa, OK 74104, USA), and the model was simplified by dropping terms that were not regarded as statistically significant (*P* > 0.05) by the analysis of variance (ANOVA). Data regarding lipase stability were processed by central tendency (mean) and dispersion (standard deviation) measurements and by the Tukey test to determine significant differences among the means. All tests were conducted in triplicate and the level of significance was 99%.

## 3. Results and Discussion

The application of lipase for transesterification reactions in organic media or in solvent-free systems has increased significantly in the last decade. Design of suitable reactors, process optimization, and the determination of effects induced by changes in operating conditions are of utter importance. Methods based on packed-bed reactors provide the best continuous way to minimize labor and overhead costs and to further develop process control to conform to commercial and industrial demands [18].

### 3.1. Immobilization of Lipase by Adsorption in a Packed-Bed Reactor Model Fitting

Lipases have two different conformations: the closed form, in which the active site is isolated from the reaction medium by a polypeptide chain (lid), is considered inactive and the open form, in which this lid is displaced and the active site is completely exposed to the reaction medium [9]. Both forms of lipases are in a conformational equilibrium affected by experimental and media conditions. In the presence of hydrophobic drops of substrate, lipases may become strongly adsorbed onto the surface of these drops, and the equilibrium is shifted towards the open form.

Compared to one-factor design, which has often been adopted in the literature, the RSM employed in this study was more efficient in reducing experimental runs and time for investigating the optimal conditions for lipase immobilization. The independent variables selected in this study were immobilization time (*t*: 1 min to 300 min), immobilization pH (pH: 1.0 to 9.0), enzyme/support ratio (ES: 1 to 1999 U/g support) and temperature (*T*: 25°C to 70°C) maintained fixed at 25°C for the immobilization process.

The experimental data were analyzed by the response surface regression (RSREG) procedure to find the best fit to the following second-order polynomial (4). The general regression equation relating independent and dependent variables is

(1) $$Y=\beta_{0}+\overset{4}{\underset{i=1}{\sum }}\beta_{i}x_{i}+\overset{4}{\underset{i=1}{\sum }}\beta_{ii}{x_{i}}^{2}+\overset{4-1}{\underset{i=1}{\sum }}\overset{4}{\underset{j=i+1}{\sum }}\beta_{ij}x_{i}x_{j},$$

where *Y* is the response (lipase activity); *β*_*o*_, *β*_*i*_, and *β*_*ij*_ (*i* = 1,2, 3,4 and *j* = 1,2, 3,4 with *j* ≥ *i*) are constant coefficients to be determined by the least squares method and *x*_*i*_ (*i* = 1,2, 3,4) are the uncoded independent variables (*x*_1_: immobilization time; *x*_2_: pH; *x*_3_: enzyme/support ratio; *x*_4_: temperature).

The best fit of (1) obtained for the experimental data shown in Table 1 for the immobilization process is

(2) $$\begin{array}{cc} Y_{\text{IE}} & =169-11.06x_{2}-65.88x_{2}^{2}\\ & \;-16.66x_{1}^{2}+33.7x_{3}\\ & \;-37.14x_{3}^{2}-15.94x_{1}x_{2}\\ & \;-19.59x_{2}x_{3}+15.37x_{1}x_{3}, \end{array}$$

where the dependence on temperature (*x*_4_) was left out, because all the data in Table 1 are for 25°C.

The ANOVA was used to evaluate the adequacy of the fitted model. The R-squared value provides a measure of the credibility of the model: values approaching 1.00 (*R* > 0.9) indicate the reliability of the model to predict the responses observed experimentally [19, 20]. The adequacy and statistical significance of the model was confirmed by the value obtained for the regression coefficient (0.88) and by the F-ratio values, since its calculated *F* value is 3.40 times higher than the critical *F* value (2.95).

**Figure 2:**
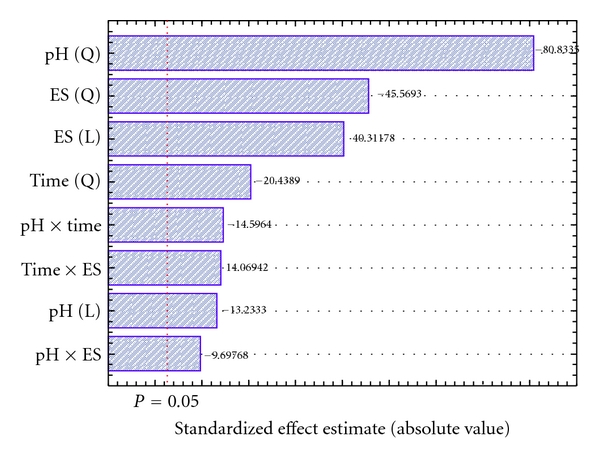
Pareto graph showing standardized effect estimates of different variables on lipase immobilization, at the CCRD.

Evaluation of the factorial design as a Pareto chart (Figure 2) demonstrates that, for the studied experimental domains, all factors are significant. The quadratic term for lipase immobilization pH presented the most pronounced standardized effect estimate on the response (−80.833), followed by quadratic and linear ES ratios (−45.5693 and 40.31178, resp.) and, less importantly, time (−20.4389). The interaction effects observed (pH and time; ES ratio and time) indicate that attempts to optimize this system using an univariate design approach would not lead to the optimal immobilization condition, since the analysis of each factor separately could not expose the combined effect of the interactions.

**Figure 3:**
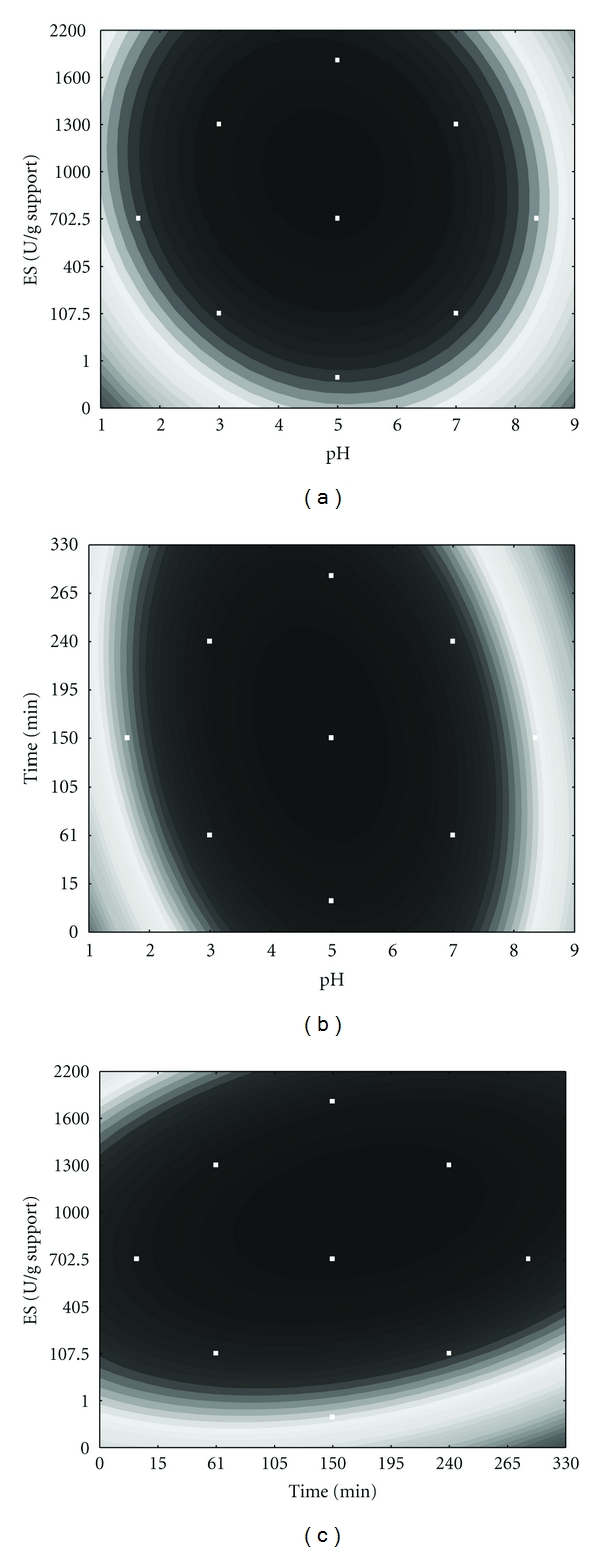
Contour diagrams for immobilized lipase activity (a) as a function of pH and enzyme/support ratio, (b) as a function of pH and time, and (c) as a function of enzyme/support ratio and time according to the first experimental design. The support was pretreated with buffer solutions (pH 1.0 to 9.0). FLCS (1–1999 U/g of support) was circulated in the column (1 min to 300 min at 25°C). After removal of unbounded lipase, the column was washed three times with 2.5 mL of buffer solution per g of support before activity measurements.

The coded model was used to generate response function contours (Figure 3) in order to analyze the effects of each of the variables on lipase activity. Figure 3 indicates that higher IE activity was achieved in the pH range from 4.0 to 6.0, with immobilization times from 1 to 240 min and with enzyme/support ratios of 1000 to 1600 U/g support. The optimal value of each variable was obtained by differentiating (2). The maximum IE activity was calculated as 177.5 U/g support at pH 4.76 with an enzyme/support ratio of 1282 U/g support for 150 min of immobilization.

The validity of the model was examined by realizing experiments at the calculated optimal activity conditions. The actual value for the IE activity was 165 U/g support, which represents 93% of the predicted value. Analyzing the effect of each independent variable on the immobilization efficiency (Figure 3), it can be observed that the lipase activity first increased significantly when the enzyme/support ratio was increased, reaching the maximum IE response at 1600 U/g support. After this point, loading more than 1600 U/g support resulted in lower enzyme immobilization. This is probably due to steric hindrance of the active site of enzyme molecules, which is either caused by the close packing of the enzymes at high concentration or by the formation of a multilayer of the adsorbed enzyme that may inhibit the access of substrate to the enzyme active site. The same effect was observed with the *Rhizopus oryzae* lipase immobilization by adsorption onto a CaCO_3_ support [21].

In our study, it was shown that pH influenced lipase immobilization by decreasing enzyme loading both at low and high pH values (Figures 3(a) and 3(b)). It is possible that low enzyme activity observed in the extremes of the pH range resulted from changes in enzyme conformation of vital importance for the enzymatic activity. The optimal pH for lipase adsorption can change depending on properties of the support. Ye et al. [1] immobilized lipase of *Candida rugosa* on a chitosan support by adsorption and found that the maximum activity was obtained with the immobilized enzyme prepared at pH 7.5.

The immobilization time, being the least important factor for *P. hubeiensis* lipase immobilization, had little influence on the optimal immobilization, which was therefore achieved in a broad range, from 1 to 300 min (Figure 3(c)). Similar results have also been reported with lipase immobilized by other methods [22–24].

The operational flexibility observed in the immobilization of lipase in a packed-bed reactor showed that this process is a good choice for industrial application.

### 3.2. Effect of Temperature and pH on Free and Immobilized Lipase Activity

Optimal conditions for maximum enzyme activity differ for free and immobilized enzymes depending on the type of the support, method of activation, and method of immobilization [25]. Therefore, the independent variables selected in this study were pH (pH: 3.0 to 9.0) and temperature (*T*: 30°C to 70°C). The ES was fixed at the optimum value of 1282 U/g support; the time of incubation was fixed at 150 min.

The general regression equation relating independent and dependent variables was fitted to the second-order model ((3) for the FLCS activity and (4) for the IE model), where *x*_4_ stands for the temperature, and the ES dependence (*x*_3_) is disregarded because it was maintained fixed:

(3) $$\begin{array}{cc} Y_{\text{FLCS}}\;\left(\text{U}/\text{mL}\;\text{supernatant}\right) & =120.9-29.35x_{2}\\ & \;-45.48x_{2}^{2}\\ & \;+30.9\;x_{4}-19.54x_{4}^{2}\\ & \;-26.64x_{2}x_{4}, \end{array}\;$$

(4) $$\begin{array}{cc} Y_{\text{IE}}\;\left(\text{U}/\text{g}\;\text{support}\right) & =69.71-26.57x_{2}^{2}\\ & \;+8.79x_{4}-23.27x_{4}^{2}. \end{array}\;$$

The ANOVA was used to evaluate the adequacy of the fitted models. The adequacy and statistical significance of the models were confirmed by the *F*-ratio values since, for the FLCS, the calculated *F* value is 7.59 times higher than the critical *F* value (4.30) and the regression coefficient (0.97) is close to unity. For the IE, the calculated *F* value is 7.55 times higher than the critical *F* value and the regression coefficient was 0.88.

**Figure 4:**
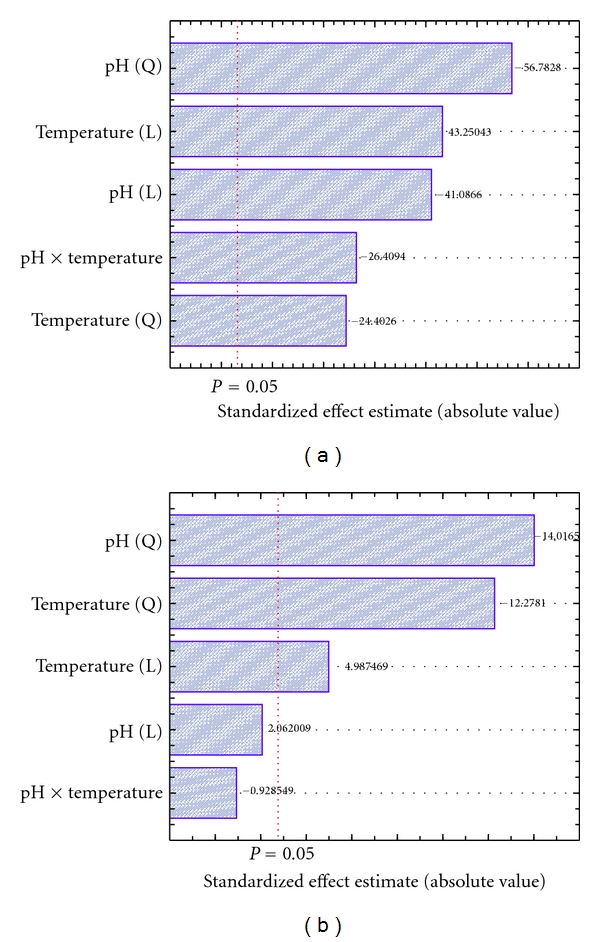
Pareto graph showing the standardized effect estimates of different pHs and temperatures on (a) free and (b) immobilized lipase activities, at the CCRD.

The Pareto chart with the standardized effect estimates of each investigated parameter is shown in Figures 4(a) and 4(b). As can be seen from Figure 4(a), the FLCS presented an expressive effect of pH (quadratic) (−56.7828) and an important effect of the interaction between pH and temperature (−26.4094) on its activity. On the other hand, in spite of the significant effect of pH and temperature (quadratic) on IE activity (−14.0165 and −12.2781, resp.), there was no significant interaction effect between pH and temperature (*P* > 0.05) on the IE activity (Figure 4(b)).

**Figure 5:**
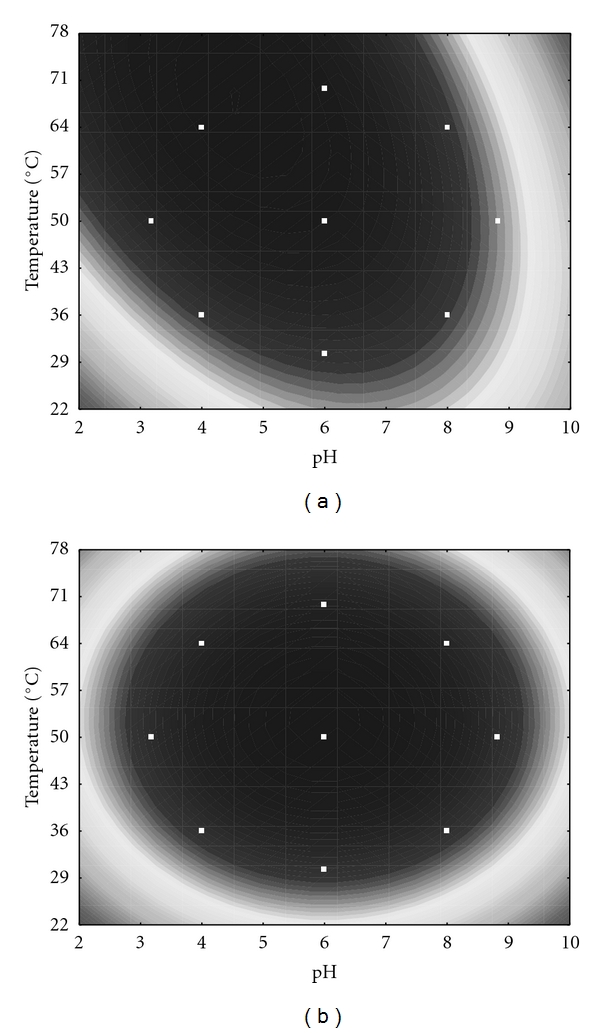
Contour diagrams for (a) free lipase and (b) immobilized lipase activities as a function of pH and temperature according to the second and third experimental designs. Optimal temperature and pH were determined in buffer solutions (pH 3.0 to 9.0) at different temperatures (30–70°C).

Repeating the analysis in Section 3.1, the coded model was again used to generate response surface contours (Figures 5(a) and 5(b)). The *P. hubeiensis* (strain HB85A) FLCS showed high activity for pH in the range from 3.0 to 6.0 and temperatures from 50 to 78°C (Figure 5(a)). The optimal value of each variable was obtained by differentiating (2) and (3). Maximal FLCS activity was 151 U/mL obtained at pH 4.6 and at 68°C.

The IE showed high activity at a broader range than the FLCS (pH 4.0 to 8.0 and temperature from 36 to 70°C). Maximal IE activity was observed at pH 6.0 and at 52°C (Figure 5(b)).

By comparing the temperature effect on the activity of FLCS and IE, it was found that the optimal temperature for the FLCS (68°C) was higher than the one for IE (52°C). Differences in the optimum temperature after immobilization have been reported by several authors [5, 9]. Several factors may be responsible for these changes, such as the three-dimensional enzyme structural changes that possibly occur during the immobilization procedure.

Although the temperature for optimal IE activity was lower than that of the FLCS, our results suggest that both enzyme forms have industrial applications under high temperature conditions. In contrast, Deng et al. [9] observed lower optimal temperatures. In their study, the optimal temperature for the free enzyme activity was 35°C and the optimal temperature for the immobilized enzyme varied, depending on which Polypropylene Hollow Fiber Membrane was used as support (40°C for 8-PAP-modified, 43°C for 12-PAP-modified and 45°C 18-PAP-modified). On the other hand, Tümtürk et al. [25] found the same optimal temperature for free and entrapped lipase, probably due to the different method of immobilization which consisted of the physical confinement of enzymes within micro spaces formed in the matrix structures of poly(*N*,*N*-dimethylacrylamide-*co*-acrylamide and poly(*N*-isopropylacrylamide-*co*-acrylamide)/k-Carrageenan hydrogels). Since in this method the enzymes do not chemically bind to the polymeric matrices they do not suffer conformational changes.

Both neutral and acidic pH showed positive effects on the activity values of FLCS and immobilized *P. hubeiensis* lipase. The pH range of the IE was slightly broader than that of the FLCS and the optimal pH increased from 4.6 in the FLCS to 6.0 in the IE. The same result has also been observed for *Candida rugosa* lipase after adsorption onto Polypropylene Hollow Fiber Membrane (pH 7.7 for the FLCS, and 8.5 for the IE) [3]. Deng et al. [9] found an optimal pH of 7.7 for the FLCS and varying optimal pH values depending on the type of the Polypropylene Hollow Fiber Membrane Modified with Phospholipid Analogous Polymers used (8.3 for 8-PAP-modified, 8.7 for 12-PAP-modified, and 8.5 for 18-PAP-modified) [26].

The validity of the model equations for the FLCS and the IE found in our work is confirmed since a relative error bellow 20% was found when the predicted activity values were compared to the experimental ones.

 Previously, we studied the effects of temperature and pH on the FLCS activity by varying one parameter while keeping the other one constant. The obtained optimal values of pH and temperature were 7.0 (60 U/mL) and 50°C (45.3 U/mL), respectively, instead of pH 4.6 and 68°C (130 U/mL) obtained in the present work. As can be seen, by using the factorial design, the maximal lipase activity increased 217% when compared to the one-way analysis [14]. The disadvantage of a single-variable optimization is that it does not reflect the interactions among the independent variables.

### 3.3. Lipase Stability

The stability of the IE, of great importance for commercial applications, depends on the strength of the noncovalent bonds formed between the support and the amino acid residues on the interacting surface of the protein.

#### 3.3.1. Effect of Temperature and pH on Lipase Stability

The thermal stability of the FLCS and the IE from *P. hubeiensis* was tested by incubation over a range of temperatures for 2 h (Table 3). The FLCS showed a good thermal stability during incubation for up to 2 h at 50°C and 60°C; at 30°C, 40°C, and 70°C a decrease in the relative activity was observed. Comparing to the FLCS, the IE presented better thermal stability at all temperatures studied, due to the fact that the interaction of lipase with the support may stabilize the conformation of the enzyme and improve the resistance of the protein to thermal denaturation [27, 28].

However, Tümtürk et al. [25] obtained lower thermal stability of the immobilized enzyme, whether it was immobilized by entrapment (23% relative activity) or by covalent bond (29% relative activity) after an incubation of 25 min at 45°C.

Our results demonstrate that both the FLCS and the IE are particularly stable at high temperatures. Since both showed better thermostability at 50°C, we chose this temperature to characterize both the FLCS and the IE with respect to other properties.

The stability of free and immobilized *P. hubeiensis* lipase was investigated over the pH range from 3.0 to 9.0 in the absence of substrate (Table 4). After 2 h at 50°C, relative activity of free and immobilized enzymes was measured under optimized conditions. Both the FLCS and the IE were stable over almost all of the pH range; however, the IE was shown to be more stable than the FLCS. This could be due to the direct interaction between the lipase and the support, which might allow the enzyme to undergo interfacial activation during immobilization, thus exposing the active site to the reaction medium. In this stabilized conformation, p-NPP hydrolysis may be facilitated.

Most lipases reported in the literature were observed to have improved stability only over specific pH ranges. Pahujani et al. [8] observed that Nylon-6 immobilized lipase was fairly stable within a pH range from 7.5 to 9.5, and the free enzyme was stable within a pH range from 8.0 to 10.5. Vaidya et al. [29] showed that immobilization of lipase from *Candida rugosa* in a macroporous polymer appreciably improved the stability at alkaline pHs. In contrast to the results of others, we demonstrate that immobilization improved lipase stability over almost all values of pH analyzed.

In spite of the FLCS having presented a high increment on relative activity at pH 7.0 after 2 h incubation (117%), the absolute activity value (10.84 U/mL) continued below that found under other pH conditions. Therefore, pH 5.0 was used for FLCS characterization because it presented both a high relative activity (83%) and a high absolute activity value (112 U/mL) after incubation for 2 h. The IE characterization was done at pH 7.0, at which its relative stability after 2 h incubation was 150%, and its absolute activity, 240 U/g support.

#### 3.3.2. Effect of Diverse Chemicals and Detergents on Lipase Activity

The effect of cations on the activity of the lipase is shown in Table 5. The IE activity showed better stability in the presence of 5 mM Mg^2+^, Ba^2+^ ions (144% and 100% relative activity, resp.) than the FLCS (98% and 7% relative activity, resp.) after 1 h incubation at 40°C. On the other hand, a reduction in the IE stability compared to the FLCS was detected in the presence of 5 mM of K^+^ ions (37% and 65% relative activity, resp.). Both the FLCS and the IE were not affected by 5 mM Zn^2+^ ions (85% and 98% relative activity, resp.) (Table 5). Lima et al. [30] observed an enhancement in the activity of the FLCS from *P. aurantiogriseum* in the presence of 1 mM Mg^2+^ ions (113% relative activity) and a reduction in the lipolytic activity of the FLCS in the presence of 1 mM of Ba^2+^ ion (70% relative activity).

We analyzed the effect of metal removal by EDTA chelating agent. EDTA reduces the FLCS activity by 45% and had no effect on the IE activity (Table 5). These results suggest that the conformation of the FLCS from *P. hubeiensis* may be modulated by cations and that immobilization stabilized the active conformation thus preventing loss of activity when incubated with EDTA (Table 5). Ca^2+^ ions enhanced the effect in the FLCS stability and reduced the IE activity. Calcium ions have been reported to form complexes with ionized fatty acids, changing their solubility and behaviors at interfaces [31]. The FLCS activity was inhibited in about 40% by 5 mM *β*-mercaptoetanol, while the activity of the IE was increased by about 40% (Table 5). Lipase from *P. hubeiensis* may contain cysteine residues that form an intramolecular disulfide bridge, and that these disulfide bonds are sensitive to reduction only in the FLCS [32]. However, the small response to *β*-mercaptoetanol suggests that there are probably no cysteine residues in the catalytic area.

It has been found that hyperactivation of lipases can be caused by detergents, which very likely stabilize their open forms by breaking the lipase homo- or heterodimers formed by interaction between the open forms of two lipase molecules [11, 33]. In our study, both the FLCS and the IE of *P. hubeiensis* were incubated for 1 h at 50°C in the presence of 1% (v/v) of various detergents (Triton X-100, Tween 80, Tween 20, and SDS). The FLCS activity was stimulated by the presence of nonionic detergents (107% with Triton X-100, 103% with Tween 80 and 123% with Tween 20), which induced major changes in the IE activity (228%, 149%, and 181% for each detergent as aforementioned) (Table 5). It is possible that, besides preventing aggregation of the lipase, the nonionic detergents stabilize the interfacial area facilitating the substrate's access to the enzyme [5]. Recently, the stabilization of the open forms of lipases adsorbed on aminated supports has been shown. Results suggested that this is a good option to obtain lipases exhibiting a higher catalytic activity [5]. However, our work shows that the anionic detergent SDS acts as a strong inhibitor in the hydrolysis activity of both the IE and the FLCS. Contrary results were observed by Cabrera et al. [33], who observed that the Triton X-100 acts as a strong inhibitor of lipase activity from *Thermomyces lanuginose* covalently immobilized on CNBr-activated agarose and that SDS increased the enzyme activity after incubation time.

#### 3.3.3. Lipase Stability in Organic Solvents

Esterification and transesterification reactions that do not occur in aqueous solutions can be carried out in organic media using enzymes. However, it is well known that enzyme activity is strongly affected by the choice of the organic solvent which may bring about the denaturation of the enzyme, thus leading to the loss of the catalytic activity [28]. In order to study tolerance of immobilized enzyme to organic solvent, the effects of various organic solvents at concentrations of 20%, 50%, and 80% (v/v) were examined (Table 6). The highest stable temperature (50°C) was chosen for the treatment of the FLCS and the IE with the different solvents. Immobilized *P. hubeiensis* lipase showed enhanced relative stability in the presence of 80% (v/v) organic solvents (101% for acetone, 77% for methanol, and 102% for 2-propanol) after 1 h incubation compared to the FLCS that retained only 25%, 17%, and 33% relative activity, respectively.

The results suggest that the support might trap and prevent the solvation of the enzyme-bound water, essential to maintain the three-dimensional structure of the enzyme for catalysis [8]. After immobilization, minor conformational changes in enzyme structure may take place, resulting in higher stability of the immobilized enzyme [34]. Such a phenomenon has also been observed by other researchers [28, 34], which means that the immobilization methods preserve the enzyme activity. On the other hand, the FLCS presented good stability in 80% (v/v) butanol and a small relative activity in 80% (v/v) ethanol (Table 6). The lipase from *B. coagulans* when immobilized on Nylon-6 presented a decrease of its lipolytic activity in the presence of methanol, ethanol, and isobutanol, and a small relative activity in the presence of acetone (15.8%) after 55 min at 30°C [8].

#### 3.3.4. Storage Stability

**Figure 6:**
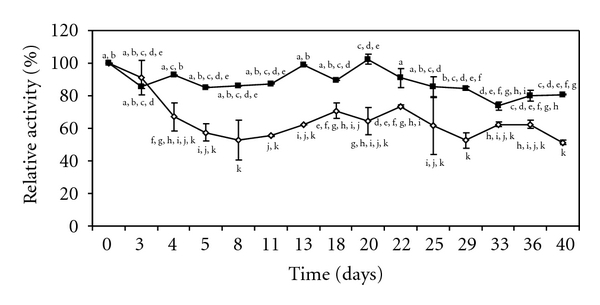
Storage stability of free (■) and immobilized (*◊*) *Pseudozyma hubeiensis* (strain HB85A) lipase. Free and immobilized enzymes were stored at 4°C. The storage stability of enzymes was tested for 40 days by determining the activity every day using the activity assay method.

Storage stability is one of the most important criteria for the application of an enzyme on a commercial scale [25]. The IE and the FLCS were stored at 4°C and activities were measured periodically over the period of 40 days. The lipase relative activity at different time intervals was estimated and results are given in Figure 6. Under the same storage conditions, the activity of the FLCS decreased at a slower rate than that of the IE (Figure 6). Upon 40 days of storage, adsorbed lipase retained about 50% of its original activity while the FLCS retained 80%. Contrary to our results, Tümtürk et al. [25] verified that covalently immobilized lipase on P(DMAm-co-AAm) and entrapped enzyme in P(NIPA-co-AAm)/Carrageenan hydrogels retained about 54% and 42.5% of their original activity, respectively. It was observed that the free enzyme lost completely its activity. Dizge et al. [10] immobilized a microbial lipase by covalent attachment onto Polyglutaralde-hydeactivated Poly(styrene-divinylbenzene) and observed that immobilized enzyme retained its full activity for 30 days in storage at 4°C. Under the same conditions, the free enzyme lost 55% of its initial activity.

## 4. Conclusion

Immobilization of enzymes is one of the most common methods to achieve their operational stability. Here we focused on lipase immobilization due to its potential application in industry. Lipase from *P. hubeiensis* was successfully immobilized by hydrophobic binding to a Polystyrene-divinylbenzene support. The optimal calculated conditions for lipase immobilization were pH 4.76, an enzyme/support ratio of 1282 U/g support, and an immobilization time of 150 min; the highest lipase activity obtained was 177.5 U/g support, in good agreement with the experimental results (165 U/g support). The optimal calculated temperature for free and immobilized enzyme activity was found to be 68°C and 52°C, respectively. Optimal calculated pH for free and IE activity was observed to be pH 4.6 and 6.0, respectively. Lipase immobilization provides enhanced enzyme activity and stability at high temperatures, at both acidic and neutral pH, and in the presence of nonionic detergents and organic solvents. Regarding the immobilization process, our results demonstrated that the continuous bioreactor model developed in this study was simple and effective, proving to be a useful technique for increasing enzymatic activity and stability, thus making this system attractive for practical applications.

## Figures and Tables

**Table 1:** Coded levels and real values (in parentheses) for the first factorial design (20 trials) and immobilized lipase activity at 25°C.

Run	pH**	*t* (min)	ES* (U/g-support)	IE* (U/g-support)
1	−1 (3)	−1 (60)	−1 (405)	2
2	+1 (7)	−1 (60)	−1 (405)	5
3	−1 (3)	+1 (240)	−1 (405)	24
4	+1 (7)	+1 (240)	−1 (405)	6
5	−1 (3)	−1 (60)	+1 (1600)	27
6	+1 (7)	−1 (60)	+1 (1600)	30
7	−1 (3)	+1 (240)	+1 (1600)	153
8	+1 (7)	+1 (240)	+1 (1600)	32
9	−1.68 (1)	0 (150)	0 (1000)	1
10	+1.68 (9)	0 (150)	0 (1000)	1
11	0 (5)	−1.68 (1)	0 (1000)	179
12	0 (5)	+1.68 (300)	0 (1000)	101
13	0 (5)	0 (150)	−1.68 (1)	1
14	0 (5)	0 (150)	+1.68 (1999)	163
15	0 (5)	0 (150)	0 (1000)	168
16	0 (5)	0 (150)	0 (1000)	165
17	0 (5)	0 (150)	0 (1000)	173
18	0 (5)	0 (150)	0 (1000)	166
19	0 (5)	0 (150)	0 (1000)	167
20	0 (5)	0 (150)	0 (1000)	171

*ES: enzyme/support ratio and IE: immobilized enzyme.

**Buffer solutions:1 M HCl for pH 1.0; 0.05 M citrate-phosphate buffer for pH 2.0, 3.0, 4.0, 5.0, 6.0, and 7.0; 0.05 M Tris-HCl buffer for pH 8.0 and 9.0.

**Table 2:** Coded levels and real values (in parentheses) for the second (12 trials) and third (13 trials) factorial design for free and immobilized lipase activity.

Run	pH**	*T* (°C)	FLCS* (U/mL supernatant)	IE* (U/g-support)
1	−1 (4)	−1 (36)	28	9
2	+1 (8)	−1 (36)	20	11
3	−1 (4)	+1 (64)	128	12
4	+1 (8)	+1 (64)	13	13
5	−1.41 (3)	0 (50)	79	12
6	+1.41(9)	0 (50)	0	35
7	0 (6)	−1.41 (30)	37	9
8	0 (6)	+1.41 (70)	145	51
9	0 (6)	0 (50)	119	74
10	0 (6)	0 (50)	122	62
11	0 (6)	0 (50)	119	74
12	0 (6)	0 (50)	123	69
13	0 (6)	0 (50)	—	69

*FLCS: free enzyme and IE: immobilized enzyme.

**Buffer solutions: 0.05 M citrate-phosphate buffer for pH 3.0, 4.0, 5.0, 6.0, and 7.0; 0.05 M Tris-HCl buffer for pH 8.0 and 9.0.

**Table 3:** Temperature stability of the free and immobilized lipase.*

Temperature	Relative activity (%)***	
	Free lipase (FLCS)	Immobilized lipase (IE)
Control**	100^d,e,f^	100^d,e,f^
30°C	50 ± 8.24^k^	102 ± 15.1^d,e^
40°C	51 ± 2.35^j,k^	91 ± 9.0^e,f,g^
50°C	85 ± 0^e,f,g,h,i^	227 ± 0^a^
60°C	87 ± 5.23^e,f,g,h^	143 ± 0^b,c^
70°C	53 ± 0^j,k^	123 ± 3.7^c,d^

*The free and immobilized enzymes were incubated at different temperatures for 2 h.

**Control: free and immobilized lipase without incubation.

***Mean values with the same letter do not statistically differ from each other by the ANOVA Tukey test (*P* = 0.01).

**Table 4:** pH stability of the free and immobilized lipase.*

pH	Relative activity (%)	
	Free lipase (FLCS)	Immobilized lipase (IE)
Control**	100^g,h,i^	100^g,h,i^
3.0	155 ± 0^e^	100 ± 6.0^g,h,i^
4.0	52 ± 9.2^l,m,n^	69 ± 13.1^j,k,l,m^
5.0	83 ± 2.1^h,i,j,k,l^	239 ± 10.1^c^
6.0	70 ± 11.6^i,j,k,l,m^	97 ± 10.8^g,h,i,j^
7.0	117 ± 13.7^f,g^	150 ± 0.1^e^
8.0	99 ± 9.2^g,h,i,j^	143 ± 5.9^e,f^
9.0	39 ± 10.9^m,n^	97 ± 7.4^g,h,i,j^

*The free and immobilized enzymes were incubated at different buffer solutions for 2 h at 50°C.

**Each pH studied had a different control. Control: free and immobilized lipase with respective buffer solution analyzed without incubation.

***Mean values with the same letter do not statistically differ from each other by the ANOVA Tukey test (*P* = 0.01).

**Table 5:** Effect of diverse chemicals and detergents on *P. hubeiensis* free and immobilized lipase activity.*

Substance	Concentration	Relative activity (%)***	
		Free lipase (FLCS)	Immobilized lipase (IE)
Control**		100	100
MgCl_2_	5 mM	98 ± 11.4^c,d^	144 ± 3.6^b^
KCl	5 mM	65 ± 8.8^d,e,f^	37 ± 6.2^f,g^
BaCl_2_	5 mM	7 ± 2.8^g^	100 ± 3.8^c^
CaCl_2_	5 mM	185 ± 11.4^a^	58 ± 4.2^e,f^
ZnSO_4_	5 mM	85 ± 4.0^c,d,e^	98 ± 3.6^c,d^
EDTA	5 mM	55 ± 0^e,f^	103 ± 7.7^c^
*β*-mercaptoethanol	5 mM	62 ± 4.4^e,f^	139 ± 11.7^b^

Triton X-100	1%	107 ± 12.4^d^	330 ± 13.6^a^
Tween 20	1%	123 ± 0^c,d^	250 ± 3.0^b^
Tween 80	1%	103 ± 10.3^d^	149 ± 6.8^c^
SDS	1%	0^e^	0^e^

*The free and immobilized enzymes were incubated in the presence of various compounds at 50°C for 1 h.

**Control: free and immobilized lipase without the addition of any substance.

***Mean values with the same letter do not statistically differ from each other by the ANOVA Tukey test (*P* = 0.01).

**Table 6:** Stability of *P. hubeiensis* free and immobilized lipase activity in organic solvents.*

Organic solvent	Concentration (%)	Relative activity (%)***	
		Free lipase (FLCS)	Immobilized lipase (IE)
Control**		100^b,c,d^	100^b,c,d^

Acetone	20	91 ± 10.3^b,c,d,e^	94 ± 6.3^b,c,d^
	50	39 ± 12.3^i,j^	102 ± 7.0^a,b,c,d^
	80	25 ± 0.6^j,k^	101 ± 1.5^b,c,d^

Methanol	20	103 ± 13.9^b,c,d,e^	169 ± 3.5^a^
	50	42 ± 8^h,i,j,k^	134 ± 5.3^b^
	80	17 ± 2.9^k,l^	77 ± 4.3^d,e,f,g^

Ethanol	20	112 ± 11.6^a,b^	85 ± 0.5^c,d,e,f^
	50	41 ± 12.5^h,i,j^	38 ± 4.3^i,j^
	80	0^k^	17 ± 11.0^j,k^

2-propanol	20	78 ± 11.1^c,d,e,f^	79 ± 0^c,d,e,f^
	50	23 ± 6.3^k,j^	110 ± 12.4^a,b,c^
	80	33 ± 8.8^i,j,k^	102 ± 6.4^a,b,c,d^

Butanol	20	77 ± 8.2^a,b,c,d^	27 ± 6.9 ^g,h,i^
	50	49 ± 5.0^d,e,f,g,h^	74 ± 11.1^b,c,d,e^
	80	72 ± 8.1^b,c,d,e,f^	59 ± 13.4^c,d,e,f,g^

*The free and immobilized enzymes were incubated in the presence of various organic solvents at 50°C for 1h.

**Control: free and immobilized lipase without the addition of any substance.

***Mean values with the same letter do not statistically differ from each other by the ANOVA Tukey test (*P* = 0.01).
